# Clinical and microbiological evaluation of foot diseases in small ruminants in Siirt province (Türkiye) and its districts

**DOI:** 10.17221/81/2024-VETMED

**Published:** 2025-02-24

**Authors:** Ali Gulaydin, Ozgul Gulaydin, Muazzez Yesilyurt, Nihat Sindak, Mustafa Baris Akgul, Onur Yildirim

**Affiliations:** ^1^Department of Surgery, Faculty of Veterinary Medicine, Siirt University, Siirt, Turkiye; ^2^Department of Microbiology, Faculty of Veterinary Medicine, Siirt University, Siirt, Turkiye

**Keywords:** CODD, *D. nodosus*, foot diseases, goat, sheep, *Treponema* spp.

## Abstract

Foot diseases are one of the leading health problems that lead to significant yield losses in small ruminant breeding. This study aimed to clinically evaluate foot diseases in sheep and goats reared in Siirt province of Türkiye and its surrounding districts. Molecular methods were used to investigate the presence of *Dichelobacter nodosus* serogroups and *Treponema* spp. phylogroups in cases with identified lesions. Clinical examination of 4 111 sheep and goats identified foot diseases in 402 animals, affecting a total of 410 feet. Contagious ovine digital dermatitis (CODD) and digital dermatitis (DD) cases were identified in 66.82% and 26.82% of diseased feet, respectively. Footrot lesions were found in 4.87% of the feet in which the disease was identified. *D.* *nodosus* was detected in 66.23% of swab samples collected from 77 CODD cases, whereas *Treponema* spp. was identified in 2.59% of the samples. Among DD cases (*n* = 110), *D. nodosus* was found in 35.45% and *Treponema* spp. in 17.27% (Group 1 = 1.81%, Group 2 = 15.45%). The majority of *D. nodosus* strains identified in the cases (*n* = 90) were classified as serogroup A (37.77%) and serogroup D (60.00%). This study revealed that CODD is a major problem in small ruminant breeding in the Siirt province and its districts. It was determined that *D.* *nodosus* serogroup A, D and *Treponema* spp*.* Group 2 strains played an important role in the aetiology of foot diseases in sheep and goats. This study represents the first comprehensive investigation of foot diseases in sheep and goats in the Siirt province and marks a significant milestone as the first study in Türkiye to identify and analyse the aetiology causes of CODD in the literature.

It has been known that foot diseases occupy an important place in small ruminant breeding when considering the loss of yield. Foot diseases cause losses in body condition, fleece yield, and milk quantity. They also lead to weak lamb births and deaths (Scott 2007).

Many factors are involved in the occurrence of foot diseases (Korkmaz and Aslan 2008). The number of animals in the breeding operation, care and feeding conditions, climatic factors, and hoof deformations can be listed among these factors (Avki et al. 2004; Yurdakul and Ozdemir 2012; In and Saritas 2014). Besides the listed predisposing factors, various pathologies can be observed in the foot region due to various bacterial (brucellosis, tuberculosis, etc.) and viral (bluetongue, foot and mouth disease, etc.) diseases (Gelasakis et al. 2013; Yurdakul 2018; Rasool et al. 2023).

Although studies to establish the aetiology of infectious foot diseases in sheep have been ongoing, bacterial foot diseases in small ruminants are clinically divided into two forms: contagious ovine digital dermatitis (CODD) and footrot. In CODD cases, purulent lesions initially appear on the dorsal coronary band of the hoof. Over time, the lesions spread towards the dead hoof and lead to detachment of the hoof capsule. Clinically, severe lameness prevails. In CODD cases, no lesion is found on the interdigital skin, unlike footrot cases (Angell et al. 2015).

Isolates of* Dichelobacter nodosus* (*D.* *nodosus*)*,* a Gram-negative bacterium, have been reported to cause footrot cases in sheep and goats (Sullivan et al. 2015; Crosby Durrani et al. 2016). *D.* *nodosus* has the ability to reproduce in anaerobic medium. The agent is divided into 10 serogroups (A–I and M) according to fimbrial antigens. The related studies have shown that there is no cross-reaction between serogroups; therefore, the vaccine prepared against one serogroup may not develop a protective immunity in animals infected with other serogroups (Benito et al. 2024).

Although *D.* *nodosus* has been found also in CODD cases, it has been reported that the disease manifests differently from footrot (Angell et al. 2015). While pathogenic *Treponema* spp. strains [*T.* *phagedenis* (phylogroup 1), *T.* *medium* (phylogroup 2), and *T.* *pedis* (phylogroup 2)] have been associated with severe cases of contagious digital dermatitis in large and small ruminants, no definite and clear information on the aetiology of the disease has yet been established (Angell et al. 2015; Sullivan et al. 2015; Crosby Durrani et al. 2016; Akkose and Izci 2017). Furthermore, although *Fusobacterium necrophorum* strains were previously known as the primary causative agent of footrot cases, recent studies have reported that this agent is an opportunistic pathogen and plays a role in aggravating the severity of clinical findings (Benito et al. 2024). It has been reported that aerobic bacterial agents are also isolated and identified as secondary agents in cases of footrot (Sertkaya and Sindak 2004).

Sheep and goat breeding occupies an important place in the animal husbandry of Türkiye and it ranks seventh in the list of sheep breeding countries, and 30–35% of meat production and 20% of milk production are met by this sector (Izci et al. 1994; Yurdakul 2018). The unfavourable climate and soil conditions for engaging in agricultural activities in Siirt province and its districts have led to small ruminant breeding among the people living there. Goods such as meat, milk, fleece, hair, mohair, and leather produced from ovine animals are among the important sources of income. According to the 2023 data of the Turkish Statistical Institute, Siirt ranks eleventh among the provinces in Türkiye, with a total number of 1 197 307 ovine animals. There has been no comprehensive study on identifying foot diseases in small ruminants in the region before. This study aimed to identify the presence of foot diseases in sheep and goats reared in the Siirt province and its districts. Along with the evaluation of clinical findings by field screening, isolation and identification of various bacteria by conventional microbiological methods and antimicrobial susceptibility results were evaluated in cases where symptoms of infection were observed. The study also investigated the presence of* D.* *nodosus* serogroups and *Treponema* spp. phylogroups in swab samples with clinically identified digital dermatitis, footrot, sinusitis interdigitalis, toe granuloma and CODD collected from small ruminants by PCR.

## MATERIAL AND METHODS

In this study, a total of 3 410 sheep and 701 goats of different breeds, ages, and sexes, which were reared in 9 farms in the city centre of Siirt province, and its Sirvan, Kurtalan, and Eruh districts between 2021 and 2022, for foot diseases were screened.

In the study, the livestock populations in the visited farms were as follows: Farm 1 housed 80 sheep (15 male and 65 female); Farm 2 contained 400 sheep (160 male and 240 female); Farm 3 accommodated 400 sheep (14 male and 386 female); Farm 4 housed 600 sheep (242 male and 358 female) and 350 goats (136 male and 214 female); Farm 5 contained 1 600 sheep (92 male and 1 508 female); Farm 6 housed 70 sheep (2 male and 68 female); Farm 7 accommodated 260 sheep (8 male and 252 female) and 240 goats (6 male and 234 female); Farm 8 housed 30 goats (6 male and 24 female); and Farm 9 housed 81 goats (2 male and 79 female). All farms were visited once during the autumn.

It was noted that the rations provided on all farms consisted of a combination of straw, barley, and wheat. Observations revealed the absence of hoof care practices, such as trimming and foot baths, across all farms. Furthermore, it was determined that the flocks were managed under a transhumant system, with the animals housed on damp soil and kept in tents.

### Ethical statement

This study was approved by the Local Ethics Committee for Animal Experiments at the Siirt University with Decision No. 2021/01/01 on 29/06/2021.

### Clinical examination

Within the scope of the study, detailed clinical examinations of sheep and goat flocks were conducted to assess foot diseases. During the diagnosis of foot diseases, the clinical evaluation criteria reported by Moore et al. (2005) for digital dermatitis (DD), Akkose and Izci (2017) for CODD, and Collins and Bowring (2020) for footrot were utilised.

The cases diagnosed with DD were classified according to the grading system reported by Moore et al. (2005). Accordingly, mild DD cases without skin irritation and bad odour were classified as Grade 1, mild DD cases with less than 5% of the affected skin and bad odour were classified as Grade 2, moderate DD cases with 5–25% of the affected skin and bad odour were classified as Grade 3, and severe DD cases with more than 25% of the affected skin and bad odour were classified as Grade 4.

The animals diagnosed with footrot were classified according to the grading system used by Moore et al. (2005) in their study. Accordingly, cases with active or healing lesion with some degree of separation at the bottom of the hoof were classified as Grade 1, cases with active lesion with significant separation at the bottom of the hoof were classified as Grade 2, cases with active lesion and extensive separation at the bottom of the hoof were classified as Grade 3, and cases with active lesion and complete separation at the bottom of the hoof were classified as Grade 4.

CODD lesions diagnosed through clinical examination were classified using the five clinical stages identified by Angell et al. (2015). In this regard, local lesions with erosive ulcerative or proliferative character (reddish and yellowish-whitish) and mild purulent discharge in the coronary band and toe skin were classified as Grade 1 CODD lesions. Dark-coloured, haemorrhagic, usually purulent, foul-smelling lesions and separation between the horn hoof and the live hoof were classified as Grade 2 lesions. Lesions with more than 50% separation of the horn hoof and necrosis of the live hoof were classified as Grade 3. When active lesions were identified despite the horn hoof growth, the lesions were classified as Grade 4. Cases in which the foot was healed and the newly grown horn hoof was found to have deformations were classified as Grade 5.

### Microbiological analysis

Swab samples were collected from cases with purulent lesions on the foot and sent to the Laboratory of the Department of Microbiology, Faculty of Veterinary Medicine, Siirt University in tubes containing transport medium and cold chain for identification of some bacterial agents by bacteriological conventional or molecular methods. For microbiological analysis, swab samples were collected from a total of 211 feet with DD (*n*** = **110), footrot (*n*** = **20), sinusitis interdigitalis (*n*** = **2), toe granuloma (*n*** = **2), and CODD (*n*** = **77) findings (Table 1). In the study, purulent discharge was rarely observed (Grade 1) in the majority of CODD cases (*n*** = **274) identified through clinical examination. Consequently, swab samples were collected from approximately 30% of the CODD cases. Care was taken to collect two swab samples from each case.

**Table 1:** Distribution of foot diseases identified in the study animals, their descriptive data, and the swab samples according to the districts

		District			
		City centre	Sirvan	Kurtalan	Eruh
**Sheep (*n* = 3 410)**					
Number of the animals examined		330	600	480	2 000
Number of the animals with lameness		34	15	33	289
Age	0–1	2	4	21	12
	1–3	17	2	12	277
	> 3	15	9	0	0
Sex	M	5	7	12	10
	F	29	8	21	279
Number of feet identified with disease	fore	15	13	22	213
	hind	19	5	13	79
**Clinically identified diseases**					
Digital dermatitis		7	16	32	30
Footrot		0	0	0	20
Sinusitis interdigitalis		1	0	1	0
Hoof deformation		0	0	1	0
Carpal arthritis		0	0	1	0
Toe granuloma		0	2	0	0
Contagious ovine digital dermatitis		26	0	0	242
Number of collected swap samples		34	18	33	100
**Goat (*n* = 701)**					
Number of animals examined		321	350	–	30
Number of animals with lameness		17	8	–	6
Age	0–1	10	0	–	0
	1–3	6	7	–	6
	> 3	1	1	–	0
Sex	M	0	7	–	1
	F	17	1	–	5
Number of feet identified with disease	fore	7	1	–	2
	hind	10	7	–	4
**Clinically identified diseases**					
Digital dermatitis		11	8	–	6
Footrot		0	0	–	0
Sinusitis interdigitalis		0	0	–	0
Hoof deformation		0	0	–	0
Carpal arthritis		0	0	–	0
Toe granuloma		0	0	–	0
Contagious ovine digital dermatitis		6	0	–	0
Number of collected swap samples		12	8	–	6

### Isolation and identification

#### BACTERIOLOGICAL CONVENTIONAL METHODS

One of the swab samples of the same case delivered to the laboratory was put into tubes containing 3 ml of sterile physiological saline, and the bacteria were transferred to the liquid phase by mixing them with a vortex. The suspension was centrifuged at 10 000** × ***g* for 5 min, and the supernatant was disposed of. The pellet at the bottom was suspended in 0.5 ml of sterile physiological saline. A 100 μl pellet of the suspension was inoculated onto blood agar medium (Oxoid, CM0271, UK), MacConkey Agar (1.05465; Merck, Germany), and Mannitol Salt Agar (CM85; Oxoid, UK) medium with 5% defibrinated sheep blood. The mediums were incubated at 37** °**C in an aerobic and 5% CO_2_ atmosphere for 24–48 hours.

At the end of the incubation period, pure cultures were obtained from the colonies formed on the medium. Isolates were stained with the Gram method, and their microscopic morphology was analysed. The isolates were then tested for oxidase and catalase and identified at the species level using commercial test kits for bacterial identification (Microgen^TM^ STREP-ID, Microgen^TM^ STAPH-ID, and Microgen^TM^ GnA + GnB-ID, Microgen^TM^ Bacillus-ID).

#### DETERMINATION OF ANTIMICROBIAL SUSCEPTIBILITY

The disc diffusion test method was used to determine the antimicrobial susceptibility of bacterial agents identified (59 *Staphylococcus* spp., 9 *Acinetobacter* spp., 4 *Moraxella* spp., 4 *Pasteurella multocida*, 3 *Escherichia coli*, 2 *Pseudomonas aeruginosa*) in accordance with the results of the identification test. Antibiotics were selected according to the bacteria species in line with the criteria reported by CLSI (2018) and EUCAST (2019) (Table 2). Test results were evaluated as susceptible, intermediate, and resistant based on the specified criteria. In the study, antimicrobial susceptibility could not be assessed as *D. nodosus* and *Treponema* spp. isolates were not cultured using microbiological methods.

**Table 2:** Antibiotic discs used to determine the antimicrobial susceptibility of the bacterial pathogens isolated in the study

Bacteria	Antimicrobial agents and evaluation criteria
*Staphylococcus* spp.	gentamicin (10 μg,), rifampin (5 μg), penicillin (10 units), cefoxitin (30 μg), cefpodoxim (10 μg), enrofloxacin (5 μg), trimethoprim + sulfamethoxazole (1.25/23.7 μg), clindamycin (5 μg), erythromycin (15 μg), tetracycline (30 μg), doxycycline (30 μg) (CLSI 2018), ciprofloxacin (5 μg) (EUCAST 2019)
*Acinetobacter* spp.	imipenem (10 μg), ciprofloxacin (5 μg), gentamicin (10 μg), tobramycin (10 μg), trimethoprim + sulfamethoxazole (1.25/23.7 μg) (EUCAST 2019)
*Moraxella* spp.	erythromycin (15 μg), trimethoprim + sulfamethoxazole (1.25/23.7 μg), tetracycline (30 μg), ciprofloxacin (5 μg), imipenem (10 μg), ertapenem (10 μg), amoxicillin + clavulanate (20/10 μg), cefotaxime (5 μg) **(**EUCAST 2019**)**
*Escherichia coli*	gentamicin (10 μg**),** streptomycin (10 μg), enrofloxacin (5 μg), tetracycline (30 μg), trimethoprim + sulfamethoxazole (1.25/23.7 μg), piperacillin + tazobactam (100/10 μg), imipenem (10 μg) (CLSI 2018), ertapenem (10 μg), ciprofloxacin (5 μg) (EUCAST 2019)
*Pseudomonas* spp.	gentamicin (10 μg), imipenem (10 μg), enrofloxacin (5 μg), streptomycin (10 μg) (CLSI 2018)
*Pasteurella multocida*	cefpodoxime (10 μg), trimethoprim + sulfamethoxazole (1.25/23.7 μg), tetracycline (30 μg), ciprofloxacin (5 μg), amoxicillin + clavulanic acid (20/10 μg) (CLSI 2018)

#### PCR ANALYSES

The presence of *D. nodosus* serogroups and *Treponema* spp. phylogroups in swab samples was identified by PCR using species-specific primers. Table 3 shows the information about the specific primers used in PCR analyses.

**Table 3:** Primer sequences and annealing temperature used for the identification of* Dichelobacter nodosus* serogroups and *Treponema* phylogroups in the swab samples using PCR

Agent	Oligonucleotide (5'-3')	Amplicon size (bp)	Annealing (°C/min)	References
*D. nodosus*	F: TCGGTACCGAGTATTTCTACCCAACACCT	783	57/1	La Fontaine et al. (1993)
	R: CGGGGTTATGTAGCTTGC			
Universal F	F: CCTTAATCGAACTCATGATTG	–	–	Dhungyel et al. (2002)
Serogroup A	R: AGTTTCGCCTTCATTATATTT	415	48/1	
Serogroup B	R: CGGATCGCCAGCTTCTGTCTT	283	52/1	
Serogroup C	R: AGAAGTGCCTTTGCCGTATTC	325	51/1	
Serogroup D	R: TGCAACAATATTTCCCTCATC	319	49/1	
Serogroup E	R: CACTTTGGTATCGATCAACTTGG	363	54/1	
Serogroup F	R: ACTGATTTCGGCTAGACC	241	51/1	
Serogroup G	R: CTTAGGGGTAAGTCCTGCAAG	279	55/1	
Serogroup H	R: TGAGCAAGACCAAGTAGC	409	51/1	
Serogroup I	R: CGATGGGTCAGCATCTGGACC	189	56/1	
*Treponema* spp. phylogroup				
Universal	F: AGAGTTTGATCCTGG R: TACCTTGTTACGACTT	1 526	40/1	Rurangirwa et al. (1999)
Group 1 (*T. medium*/*T. vincentii*-like)	F: GAATGCTCATCTGATGACGGTAATCGACG R: CCGGCCTTATCTAAGACCTTCTACTAG	475	61/1	Evans et al. (2009)
Group 2 (*T. phagedenis*-like)	F: GAAATACTCAAGCTTAACTTGAGAATTGC R: CTACGCTACCATATCTCTATAATATTGC	400	56/1	
Group 3 (*T. denticola*/*T. putidum*-like)	F: GGAGATGAGGGAATGCGTCTTCGATG R: CAAGAGTCGTATTGCTACGCTGATATATC	475	60/1	

#### DNA ISOLATION

A commercial DNA isolation kit (MG-BSDNA-01-100; Hibrigen, Türkiye) was used to produce genomic DNA from swab samples. The produced genomic DNA was stored at –20 °C until used in PCR procedures.

#### AMPLIFICATION

The conventional PCR method was used to identify the presence of *D. nodosus* and its serogroups in swab samples. Therefore, a commercial mastermix (2X PCR Mastermix; ABT^**®**^, Ankara, Türkiye) was used to prepare the PCR mixture. For optimisation of the mixture, 5 μl of genomic DNA and 1.5 μl each of primers (10 μM) were added to 12.5 μl of mastermix, and the total volume was topped up to 25 μl with PCR water. The amplification process was optimised according to the recommendations of the company that produces the mastermix and primers. For this reason, the PCR mixture was kept at 94 °C for 10 min for pre-denaturation. In the amplification step of a total of 35 cycles, there was a denaturation at 94 °C for 1 min and an elongation at 72 °C for 1 minute. The final elongation was set at 72 °C for 15 minutes. DNA-free PCR water was used as negative control in the tests.

The Nested PCR method as reported by Evans et al. (2009) was used to identify *Treponema* spp. phylogroups from the collected swab samples (Table 3). Accordingly, DNA samples were first subjected to PCR analysis with universal primers and then the produced amplicons were subjected to PCR procedures with Group 1, Group 2, and Group 3 primers, respectively. The PCR mixtures were prepared, and amplification procedures were performed as described for identifying the presence of *D. nodosus* in the samples by PCR.

#### ELECTROPHORESIS

The amplicons produced at the end of PCR procedures were electrophoresed (80 V, 1.5 h) in agarose gel containing gel red. Amplicons were compared with DNA markers (100 bp Plus Opti-DNA Marker G193; ABM, Richmond, Canada) and analysed on a gel imaging system (Gen-Box ImagER, Ankara, Türkiye).

### Statistical analysis

Fisher’s Exact test method was used for the statistical analysis. The value of *P* ≤ 0.05 was accepted as statistically significant.

## RESULTS AND DISCUSSION

The number of animals clinically examined during the study, the number of animals diagnosed with foot diseases, the distribution of animals by age and sex, and the distribution of swab samples collected are presented in Table 1. As a result of clinical examination, foot disease was identified in a total of 402 animals (9.77%). The number of feet with foot disease was 410. While 371 (92.28%) of the animals with foot disease were sheep, 31 (7.71%) were goats. The prevalence of foot disease was higher in sheep than in goats (*P* < 0.05).

**Figure 1:**
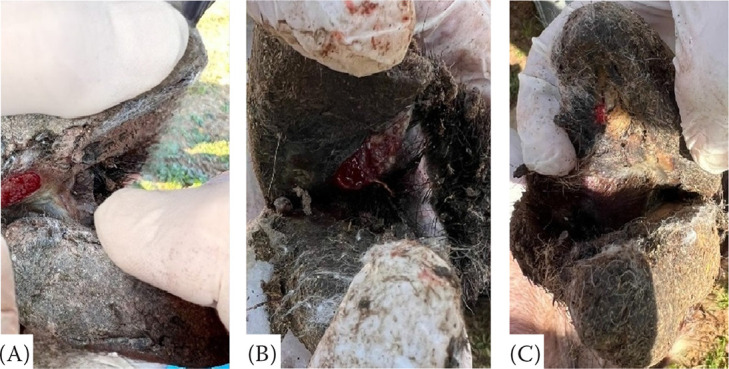
Images of contagious ovine digital dermatitis lesions (A) Contagious ovine digital dermatitis Grade 1; (B) Contagious ovine digital dermatitis Grade 2. (C) Contagious ovine digital dermatitis Grade 3

**Figure 2:**
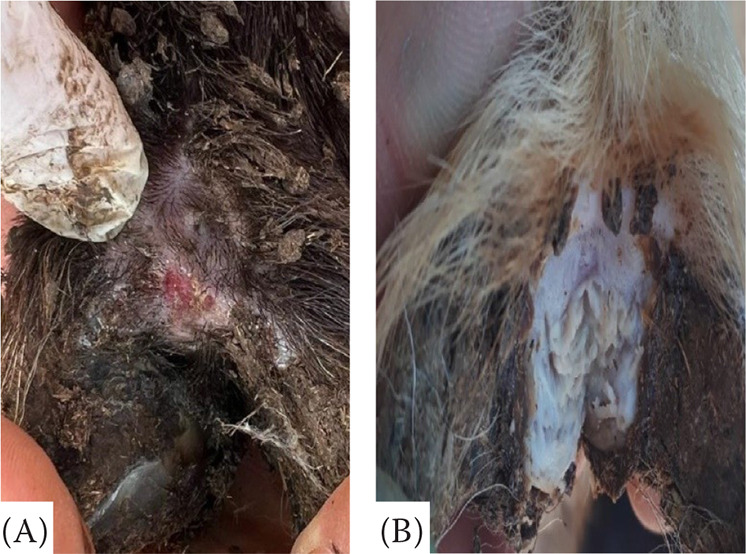
Images of digital dermatitis lesions (A) Digital dermatitis Grade 2; (B) Digital dermatitis Grade 3

**Figure 3:**
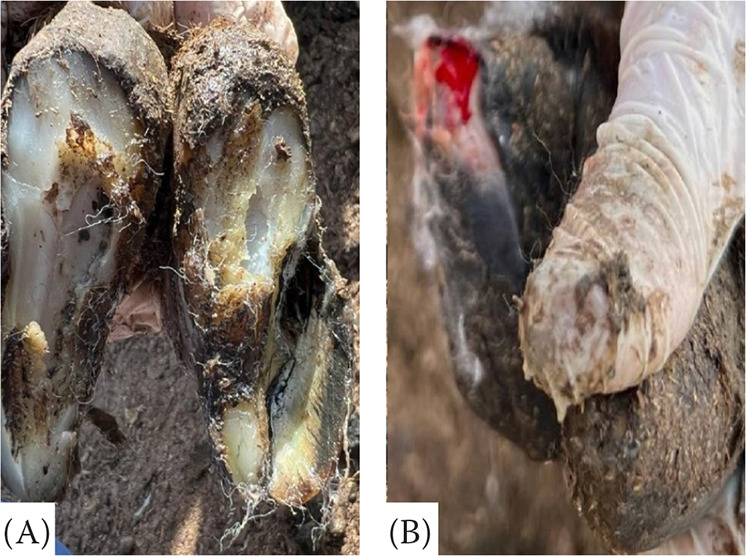
Images of footrot (A) and toe granuloma (B) lesions

When DD lesions were graded, it was determined that 17 (15.45%), 40 (35.36%), 50 (45.45%), and 3 (2.72%) of the cases were Grade 1, 2, 3, and 4, respectively. Grade 1, 2, and 3 footrot lesions were identified in 3 (15.00%), 5 (25.0%), and 12 (60.0%) cases diagnosed with footrot in the examined feet, respectively. When the lesions identified as CODD were analysed, Grade 1, Grade 2, and Grade 3 CODD findings were observed in 176 (64.23%), 45 (16.42%), and 53 (19.34%) of the cases, respectively (Figures 1, 2 and 3).

**Figure 4:**
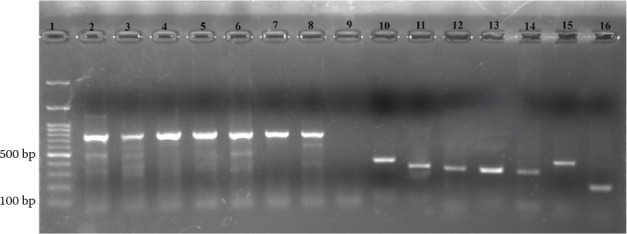
Agarose gel image of amplicons obtained from swab samples by PCR 1: 100 bp marker, 2–8: Amplicons positive for *D. nodosus* (783 bp), 9: Negative control, 10: Amplicon positive for *D. nodosus* serogroup A (415 bp), 11: Amplicon positive for *D. nodosus* serogroup C (325 bp), 12: Amplicon positive for *D. nodosus* serogroup B (283 bp), 13: Amplicon positive for *D. nodosus* serogroup D (319 bp), 14: Amplicon positive for *D. nodosus* serogroup G (279 bp), 15: Amplicon positive for *D. nodosus* serogroup H (409 bp), 16: Amplicon positive for *D. nodosus* serogroup I (189 bp)

**Figure 5:**
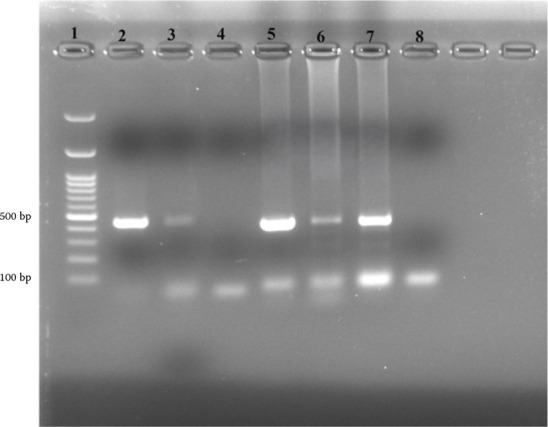
Agarose gel image of amplicons obtained from samples positive for *Treponema* spp. by PCR 1: 100 bp DNA marker, 2–3: Amplicon positive for *Treponema* spp. Group 1 (475 bp), 4: Negative control for *Treponema* spp. Group 1, 5–7: Amplicon positive for *Treponema* spp. Group 2 (400 bp), 8: Negative control for *Treponema* spp. Group 2

The presence of *D. nodosus* and *Treponema* spp. was investigated by PCR in the swab samples (Figures 4, 5). Accordingly, *D. nodosus* was identified in 39 (35.45%) of 110 DD cases, *Treponema* spp. Group 1 in 2 (1.81%), and *Treponema* spp. Group 2 in 17 (15.45%) cases. Distribution of *D. nodosus* and *Treponema* spp. phylogroups in cases from which swab samples were collected is shown in Table 4.

**Table 4:** Distribution of *D. nodosus* and *Treponema* spp. phylogroups in cases from which swab samples were collected

Agent identified by PCR	Sheep			Goat	
	digital dermatitis (*n*** = **85)	CODD (*n*** = **76)		digital dermatitis (*n*** = **25)	CODD (*n*** = **1)
*D. nodosus*	31 (36.47%)	51 (67.10%)		8 (32.0%)	0
*Treponema* spp. Group 1	2 (2.25%)	0		0	0
*Treponema* spp. Group 2	13 (15.29%)	2 (2.63%)		4 (16.0%)	0
*D. nodosus* + *Treponema* spp. Group 1	1 (1.17%)	0		0	0
*D. nodosus* + *Treponema* spp. Group 2	10 (11.76%)	2 (2.63%)		4 (16.0%)	0

CODD = contagious ovine digital dermatitis

Following the identification of *D. nodosus* in samples collected from sheep and goats, serogroups of the agent were identified based on the districts from which samples were collected by PCR using group-specific primers. The evaluation revealed that *D. nodosus* serogroup A caused foot diseases in samples collected from sheep in the city centre of Siirt province, and *D. nodosus* serogroup A and serogroup D strains caused foot diseases in Kurtalan district. Six *D. nodosus* strains identified in samples collected from Sirvan district could not be serogrouped by PCR. On the other hand, while serogroup B strains of *D. nodosus* were identified in samples collected from goats in the city center, *D. nodosus* strains identified in Sirvan district belonged to serogroups C, G, H, and I (Figure 4). In the study, in 57 (27.01%) of the 211 swab samples the agent was not isolated, while 14 (6.63%) of the samples were contaminated with *Proteus* spp. A total of 122 Gram-positive and 22 Gram-negative bacteria were isolated from the samples where growth was observed. 59 (48.36%) of the Gram-positive bacteria were identified as *Staphylococcus* spp., 8 (6.55%) as *Bacillus* spp., and 55 (45.08%) as Gram-positive irregular bacilli.

Using a commercial identification test kit, 5 of the *Staphylococcus* spp. isolates were identified as *S. auricularis*, 1 as *S. capitis* subsp. *capitis*, 3 as *S. epidermis*, 18 as *S. haemolyticus*, 10 as *S. hyicus*, 1 as *S. lentus*, 1 as *S. schleiferi* subsp. *schleiferi*, 1 as *S. sciuri*, 10 as *S. simulans*, 3 as *S. vitulinus*, 1 as *S. warneri*, and 5 as *S. xylosus.* 1 of *Bacillus* spp. isolates was identified as *B. circulans*, 1 as *B. lentus*, 1 as *B. licheniformis*, and 5 as *B. pumilus*. Since Gram-positive bacterial agents with irregular bacilli morphology are generally identified by molecular methods, they could not be identified at the species level with the commercial identification test kits used in the study. However, 9 of the Gram-negative isolates were identified as *Acinetobacter* spp., 3 as *E. coli*, 4 as *Moraxella* spp., 2 as *P. aeruginosa*, and 4 as *P. multocida*.

All *Staphylococcus* spp. isolates (*n*** = **59) were susceptible to gentamicin, rifampin, sulfamethoxazole + trimethoprim, and enrofloxacin. However, 86.44%, 89.83%, 83.05%, 49.15%, 32.20%, 35.59%, 42.36%, and 98.30% of the isolates were susceptible to penicillin, cefoxitin, cefpodoxime, clindamycin, erythromycin, tetracycline, doxycycline, and ciprofloxacin, respectively.

While all *Acinetobacter* spp. isolates (*n*** = **9) were susceptible to imipenem, 88.88% of them were susceptible to gentamicin, 77.77% to sulfamethoxazole + trimethoprim, 44.44% to ciprofloxacin, and 77.77% to tobramycin.

While 33.34% of *E. coli* isolates were found to be resistant to tetracycline and streptomycin, all strains (*n*** = **3) were susceptible to gentamicin, enrofloxacin, sulfamethoxazole + trimethoprim, ciprofloxacin, imipenem, ertapenem, and piperacillin + tazobactam.

*Moraxella* spp. isolates were highly resistant to erythromycin (100%) and sulfamethoxazole + trimethoprim (75%), while their resistance to tetracycline (25%) was low. However, all isolates (*n*** = **4) were susceptible to ciprofloxacin, imipenem, ertapenem, amoxicillin + clavulanic acid, and cefotaxime.

*P. aeruginosa* and *P. multocida* isolates were susceptible to the antibiotics used (gentamicin, enrofloxacin, streptomycin, imipenem, and amoxicillin + clavulanic acid; cefpodoxime, sulfamethoxazole + trimethoprim, tetracycline, ciprofloxacin, amoxicillin + clavulanic acid, and cefotaxime, respectively).

Different studies have reported that the incidence of foot diseases in small ruminants varies between 2.62% and 25.29% (Avki et al. 2004; In and Saritas 2014; Yurdakul 2018; Polat 2022). On the other hand, this study showed that 9.77% of the sheep and goats clinically examined had foot disease. This rate was within the limits set in previous studies conducted in Türkiye. In studies conducted on foot diseases in goats, Anzuino et al. (2010), Groenevelt et al. (2015), and Crosby Durrani et al. (2016) reported the rate of foot diseases as 19.2%, 12–67%, and 24%, respectively. This study revealed that the rate of goats with foot disease was 4.42%. The study conducted by Celebi et al. (2016) on 10 sheep herds in Kars province determined the incidence of foot disease to be 17.07%. This study showed that sheep’s foot disease rate was 10.87%. The differences in the rates determined in the studies were considered to be associated with differences in geographical regions, seasonal conditions, and care practices.

Studies have reported that foot diseases are identified more in female animals than in males (Sagliyan et al. 2003; Avki et al. 2004; In and Saritas 2014; Yurdakul 2018). This has been reported to be associated with the diligent care of the breeders for the female animals they breed, the proportionally lower number of male animals for breeding in the herds, and the delivery of the other male animals to slaughter (In and Saritas 2014; Yurdakul 2018). Likewise, the present study showed most (89.55%) animals with foot disease were females.

The literature review indicated that foot disease lesions were found more frequently in the forefeet (Bokko and Chaudheri 2001; In and Saritas 2014), and studies are reporting that lesions were detected more frequently in the hind feet (Izci et al. 1994; Sagliyan et al. 2003). This study, where 273 (66.58%) of the lesions were found in the forefeet and 137 (33.41%) in the hind feet, was compatible with the data gathered by Bokko and Chaudheri (2001) and In and Saritas (2014).

Studies conducted in different regions of Türkiye have reported that non-infectious hoof deformations are frequently observed in sheep (Polat 2022). Accordingly, the incidence rates of non-infectious hoof deformations in sheep varied between 53.13–82.52% (Avki et al. 2004; In and Saritas 2014; Yurdakul 2018; Polat 2022). This study conducted in the Siirt province and its districts showed that 0.24% of the animals had hoof deformation. The data gathered were quite low compared to those reported by previous studies. It is thought that this may be associated with the fact that most farms in Siirt province and its districts are located in mountainous, hard-packed terrain and animals need to walk long routes to reach the pastures.

Studies conducted in various regions of Türkiye (Afyon, Sivas, Kars, Burdur, and Elazıg) have reported that the prevalence of footrot in sheep and goats ranges from 6.49% to 30.99% (Avki et al. 2004; In and Saritas 2014; Baran et al. 2015; Yurdakul 2018; Polat 2022). The prevalence of the disease has been reported as 12.54% (Rather et al. 2011) and 16.41% (Farooq et al. 2010) in studies conducted in Kashmir, India. In the United Kingdom, the prevalence was reported as 10% (Wassink et al. 2003), while in Bhutan, it was reported as 3.1% (Gurung et al. 2006). In this study, the prevalence of footrot in the examined animals was determined to be 4.87%.

The prevalence of digital dermatitis (DD) in studies conducted in Türkiye’s Sivas, Kars, and Elazıg regions was found to range between 5.88% and 12.46% (Baran et al. 2015; Yurdakul 2018; Polat 2022). However, in the current study, the prevalence of DD was determined to be 26.82%.

The variability in disease detection rates among studies may be attributed to geographical and climatic differences between regions, as well as differences in farming practices, animal care and feeding conditions, and the number of animals included in the studies.

In 1997, the United Kingdom first described CODD as a major foot disease in sheep (Harwood et al. 1997; Duncan et al. 2021). Current epidemiological data suggest that CODD develops on approximately 50% of farms in the UK (Angell et al. 2014; Dickins et al. 2016; Duncan et al. 2021), and the on-farm prevalence ranges from 2% to 50% (Angell et al. 2014; Duncan et al. 2021). The disease has also been reported in countries such as Ireland (Sayers et al. 2009), Germany (Tegtmeyer et al. 2020), and Sweden (Duncan et al. 2021). In a study conducted in Türkiye, 102 (54.5%) lambs and 68 (30%) sheep were affected by CODD in a herd with 410 animals (187 lambs and 223 ewes) diagnosed with CODD based on clinical findings (Alkan et al. 2016). In this study, conducted for the first time in the Siirt province and its districts, CODD was identified at a rate of 66.82%.

While the findings showed the prevalence and severity of the disease, it was considered that more studies on the spread and effects of CODD in Türkiye are of critical importance in the management of the disease.

Studies have emphasised that the most important predisposing factor in the occurrence of CODD cases is footrot (Angell et al. 2015). However, it has not been fully clarified whether there is a synergy between CODD and footrot, whether *D. nodosus* strains play a role in the aetiology of CODD, or whether both diseases occur due to similar environmental factors (Angell et al. 2015; Akkose and Izci 2017). Interviews with owners of the farms and anamnesis information during fieldwork in Siirt province and its districts where the present study was conducted revealed that severe cases of footrot and DD were common in sheep and goats in the past. Furthermore, it was considered that the pastures commonly used by cattle and small ruminants that migrate to cooler regions due to the extremely hot climate of Siirt province and its districts during the summer also contributed to the spread of CODD.

Although the aetiology of CODD cases has not been fully clarified, it has been known that spirochetes such as *Treponema* spp. play an important role in these cases (Sayers et al. 2009). Several studies reported that *D. nodosus* and *F. necrophorum* strains were also isolated from CODD cases, but the role of these bacteria in the pathogenesis of the disease has not been fully established (Duncan et al. 2014). Benito et al. (2024) reported that *D. nodosus, F. necrophorum,* and pathogenic *Treponema* phylogroups may coexist in foot dermatitis and necrosis in small ruminants. Rasool et al. (2023) identified only *D. nodosus* in 27.4% and both *D. nodosus* and *F. necrophorum* in 54.4% of the samples collected from cases diagnosed with CODD. The researchers stated that the synergistic effect of the agents intensified the severity of the lesions. They also found that strains of *D. nodosus, F. necrophorum,* and *Trueperella pyogenes* were isolated together with *Treponema* spp. in advanced stages of CODD lesions (Grade 4), while cases in which *Treponema* spp. strains were isolated alone or with any other bacteria presented with milder clinical findings (Grade 2–3 CODD). The present study showed that *D. nodosus* was identified in 51 CODD cases, only *Treponema* spp. Group 2 in 2 cases, and both *D. nodosus* and *Treponema* spp. Group 2 in 2 cases. The findings of Grade 1 CODD were observed in 64.23%, Grade 2 in 16.42%, and Grade 3 in 19.34% of the cases. Similar to the data reported by Rasool et al. (2023), the detection of *D. nodosus* alone in most of the CODD cases in this study and the diagnosis of the agent in combination with *Treponema* spp. in only 2 cases were considered to be compatible with the clinical classification of the majority of the cases as Grade 1.

In sheep flocks in Sweden, *Treponema* spp*.* was identified in 89.7%, 94.7%, and 100% of animals with foot rot scores of 0, 1, and 2, respectively (Rosander et al. 2023). In their study on CODD in sheep in the United Kingdom, Sullivan et al. (2015) detected *Treponema* spp*.* Group 1, *Treponema* spp. Group 2, and *Treponema* spp. Group 3 strains in 67%, 85%, and 71% of 58 samples, respectively, using PCR. Rasool et al. (2023), in their study in the Kashmir region, reported that pathogens of the *Treponema* genus were identified in 48% of samples taken from 254 small ruminants diagnosed with CODD. In this study, *Treponema* spp. was detected in 17.27% of DD cases (*n*** = **19) and 2.59% of CODD cases (*n*** = **2). Similar to the data obtained by Sullivan et al. (2015), most strains were identified as agents in Group 2. The rates of *Treponema* spp. in the study were low compared to other studies. Information in the literature (Rasool et al. 2023) indicates that *Treponema* spp. is more prevalent when the clinical grading of CODD rises, both alone and as mixed infections. However, the present study graded CODD lesions from 1 to 3, which may explain both the low rate of *Treponema* spp. diagnosis and the low rate of mixed infection with other bacteria. These findings support the effect of CODD clinical grading on the intensity and diversity of infection.

In their study covering footrot in sheep herds in Sweden, Rosander et al. (2023) found that the prevalence of *D. nodosus* was 9.1% on a herd basis and 5.7% on an animal basis. Storms et al. (2021) reported a prevalence of *D. nodosus* of 42.93% in samples taken from lesions caused by footrot in German sheep flocks. A study conducted by Celebi et al. (2016) on footrot in sheep in the Kars region of Türkiye in 2016 reported that 153 (78.46%) of 195 isolates analysed by PCR were identified as *D. nodosus*. In this study, *D. nodosus* was identified in 35.45% of DD and 66.23% of CODD cases. This difference in *D. nodosus* rates may be attributed to the differences in geographical and care conditions, genetic predisposition of animals, and diagnostic methods used.

In a study conducted in the Extremadura region of Spain, 40.7% and 25.9% of *D. nodosus* strains were identified as serogroup A and serogroup C, respectively (Lacombe Antoneli et al. 2006). A study conducted in the Jammu and Kashmir region of India reported that serogroup A was not found, while the identification rate of serogroup B was 78.7% (Wani et al. 2019). A study conducted in 83 farms in Germany reported that serogroup A was the most prevalent one (38.1%), followed by serogroup B (31.1%), serogroup H (21.4%), and serogroup G (6.25%) (Albuquerque et al. 2022). A study conducted by Benito et al. (2024) in Spain and Portugal indicated that *D. nodosus* serogroup B and D strains were identified more prevalently. The serogroups of *D. nodosus* strains isolated in studies conducted in different regions differed. It is critical in identifying the serogroups of the isolates for preventive medicine on a regional basis. This study showed that *D. nodosus* isolates mostly had serogroup A antigen. Furthermore, serogroups B, C, D, G, H, and I were identified in the study. The data contributed to the protection-control programmes to be established against the disease.

The uncontrolled use of antimicrobial agents in sheep and goat farming negatively affects animal, human, and environmental health, contributing to the rapid spread of resistant bacteria. In the presented study, the antimicrobial susceptibility results of bacteria isolated as secondary pathogens from the cases were also evaluated against antimicrobial agents classified in categories A and B by the Antimicrobial Advice Ad Hoc Expert Group (AMEG). In this context, it was determined that the pathogens could also be susceptible to antimicrobial agents such as rifampin, enrofloxacin, third-generation cephalosporins, imipenem, and piperacillin + tazobactam, which are not recommended for use in livestock. However, the data obtained are included solely to contribute to the literature, and the use of these antimicrobial agents in sheep and goat farming is not recommended.

This study comprehensively and clinically identified the foot diseases that cause significant yield losses in sheep and goat herds reared in Siirt province and its districts and graded the identified cases. DD, and footrot cases, especially CODD, were found to be important diseases in small ruminant breeding in the study region. Besides the clinical evaluation of the diseases, the aetiology of infectious diseases was also identified, and it was found that *D. nodosus* serogroup A, D, and *Treponema* spp. Group 2 strains were the most important bacterial agents causing foot diseases in the study region. Furthermore, it was determined that *Staphylococcus* spp. isolates were the leading bacterial agents isolated as secondary bacterial agents in foot diseases in sheep and goats. It was found that antimicrobial agents such as sulfonamids (categorised in class D) and aminoglycosides (categorised in class C) can be used for the effective treatment of secondary bacterial agents in foot diseases. Furthermore, in this study, conducted for the first time in Siirt province and its districts, CODD was identified at a rate of 66.82% on a herd basis in Türkiye. The findings showed the prevalence and severity of the disease and that further studies on the spread and effects of CODD are critical in managing the disease. Furthermore, it is believed that farmers’ awareness of proper hoof care and foot baths should be increased in sheep and goat farming operations in the study region. It was concluded that the data would contribute significantly to small ruminant breeding, animal health, and the clinical and epidemiologic evaluation of foot diseases.
